# Cobalt/Iron Bimetallic Biochar Composites for Lead(II) Adsorption: Mechanism and Remediation Performance

**DOI:** 10.3390/molecules29071595

**Published:** 2024-04-03

**Authors:** Jingyu Zhao, Yuhong Qin, Yue Liu, Yunlong Shi, Qiang Lin, Miao Cai, Zhenya Jia, Changjiang Yu, Anqi Shang, Yuxiao Fei, Jiayi Zhang

**Affiliations:** 1 Key Laboratory of Water Pollution Treatment & Resource Reuse of Hainan Province, Key Laboratory of Natural Polymer Function Material of Haikou City, College of Chemistry and Chemical Engineering, Hainan Normal University, No. 99 Longkunnan Road, Haikou 571158, China; 2 Hainan Pujin Environmental Technology Co., Ltd., Haikou 570125, China; 3 Hainan Huantai Environmental Resources Co., Ltd., Haikou 571158, China

**Keywords:** nano-zero-valent iron, cobalt, biochar, lead(II) adsorption, remediation

## Abstract

The performance of nano-zero-valent iron for heavy metal remediation can be enhanced via incorporation into bimetallic carbon composites. However, few economical and green approaches are available for preparing bimetallic composite materials. In this study, novel Co/Fe bimetallic biochar composites (BC@Co/Fe-X, where X = 5 or 10 represents the CoCl_2_ concentration of 0.05 or 0.1 mol L^−1^) were prepared for the adsorption of Pb^2+^. The effect of the concentration of cross-linked metal ions on Pb^2+^ adsorption was investigated, with the composite prepared using 0.05 mol L^−1^ Co^2+^ (BC@Co/Fe-5) exhibiting the highest adsorption performance. Various factors, including the adsorption period, Pb^2+^ concentration, and pH, affected the adsorption of Pb^2+^ by BC@Co/Fe-5. Further characterisation of BC@Co/Fe-5 before and after Pb^2+^ adsorption using methods such as X-ray diffraction and X-ray photoelectron spectroscopy suggested that the Pb^2+^ adsorption mechanism involved (i) Pb^2+^ reduction to Pb^0^ by Co/Fe, (ii) Co/Fe corrosion to generate Fe^2+^ and fix Pb^2+^ in the form of PbO, and (iii) Pb^2+^ adsorption by Co/Fe biochar. Notably, BC@Co/Fe-5 exhibited excellent remediation performance in simulated Pb^2+^-contaminated water and soil with good recyclability.

## 1. Introduction 

Currently, Pb pollution is among the most serious environmental issues. Pb enters the human body through the food chain and can detrimentally affect the central nervous system, liver, and kidneys [1]. Common methods for Pb removal include chemical precipitation, ion exchange, membrane filtration, adsorption, and electrochemical treatment [2]. Adsorption is the most widely used treatment technology owing to its low cost and high efficiency. Typical adsorbents consist of carboxymethyl cellulose/graphene oxide/Fe nanoparticles [2], modified chitosan hydrogels [3], analcime-activated carbon composites [4], nano-zero-valent iron (nZVI) [5], and amino-carboxyl cellulose [6]. nZVI has been extensively used in the remediation of hazardous groundwater, permeable reactive barriers, and contaminated subsoil [5,7]. Although nZVI has many benefits for treating heavy metal pollution [8], including high mobility, high reactivity, excellent reducibility, and a large specific surface area, issues such as easy passivation in air and easy agglomeration limit its applicability to environmental remediation [9]. Enhanced stability and higher adsorption capacities have frequently been achieved via composite formation or surface modification. The combination of nZVI with porous carbon materials such as carbon nanotubes [10], activated carbon [11], and biochar [12,13], which have unique pore structures, good chemical stability, and high electrical conductivity, can effectively reduce agglomeration and increase pollutant removal efficiency [14]. In particular, bimetallic carbon composites are considered promising for improving the reactivity of nZVI.

Xing et al. [15] prepared a novel mesoporous Santa Barbara Amorphous-15-supported Fe/Ni bimetallic composite (SBA-15@Fe/Ni) to remove Cr(VI). Their results showed that the removal efficiency of SBA-15@Fe/Ni is better than two separate systems (SBA-15 and Fe/Ni). Introducing Ni can promote Cr(VI) reduction through electron transfer and catalytic hydrogenation. Yuan et al. [16] prepared microscale iron–copper (mFe/Cu) bimetals wherein copper coated the surface of mFe^0^ particles. Electrochemical analysis revealed that copper plating promotes the release of electrons from mFe^0^ and reduces the impedance of mFe^0^. Fe and Co reportedly have synergistic catalytic effects; a low percentage of Co can improve the reduction activity of Fe [17]. However, research on contaminant removal by Fe/Co composites has rarely been reported [18]. Qin et al. [19] prepared FeCo bimetallic nanoparticles via a hydrothermal reduction method, which can achieve higher activity and significantly improve the reaction kinetics for removing Cr(VI) compared with those of Fe^0^. Hong et al. [20] prepared a novel 2D metal–organic complex that exhibits highly efficient removal performance for trace Pb^2+^ in neutral aqueous solutions (pH 7).

The primary technique for preparing bimetallic composites is liquid-phase reduction, in which metal ions such as Cu^2+^ and Ni^2+^ are reduced using NaBH_4_ and loaded onto the surface of Fe. However, the NaBH_4_ reduction process is expensive and produces numerous secondary pollutants [21]. In addition, Fe/Co bimetals prepared by hydrothermal methods generally exist as alloys and have low activity when treating pollutants. Therefore, developing economical and green methods for preparing bimetallic composite materials is highly desirable. The carbothermal method has recently gained popularity as an environmentally friendly approach for preparing Fe/C composites [22]; zero-valent metals were produced during the carbothermic reduction process and loaded onto biochar [23]. Nonetheless, carbon-supported bimetallic nanoparticles synthesised using this method have rarely been reported.

Sodium alginate (SA), a renewable natural resource, contains a large number of carboxyl and hydroxyl groups capable of interacting with divalent (e.g., Ca^2+^) or trivalent (e.g., Fe^3+^) ions to form hydrogels [24]. Motivated by this property, SA-Fe^3+^/Co^2+^ gels were prepared and used as precursors for carbothermal reduction synthesis, with Co/Fe-embedded biochar (BC@Co/Fe-X, where X represents the Co^2+^ concentration during gel preparation) produced via one-step pyrolysis. This synthesis has several advantages: (ⅰ) compared with the traditional metal salt impregnation method, SA-Fe^3+^/Co^2+^ gels, as precursors, can better ensure the dispersion of metal ions; (ⅱ) Fe^3+^(Co^2+^)–O clusters were isolated by ligands in the complex and then reduced in situ to bimetallic Co/Fe nanoparticles, which ensured their stability; and (ⅲ) ligands were pyrolysed to biochar, which provides conductive and large surface area support to immobile nanoparticles. 

Therefore, the objective of this study was to prepare efficient bimetallic composite materials using a hydrothermal reduction method and investigate the adsorption mechanisms for Pb^2+^. The effect of Co doping on the Pb^2+^ adsorption performance of the composites was analysed. The obtained BC@Co/Fe-X composites exhibited a large surface area, well-designed structure, and efficient adsorption performance. Furthermore, the Pb^2+^ adsorption mechanism of BC@Co/Fe-5 and its remediation performance in Pb^2+^-contaminated water and soil were investigated.

## 2. Results and Discussion

### 2.1. Characterisation

**Figure 1:**
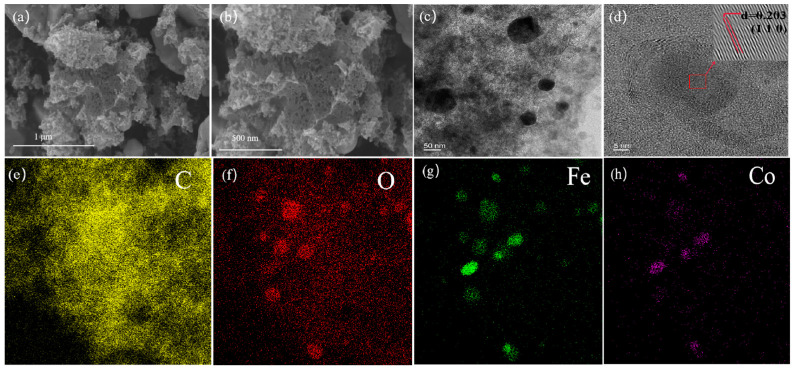
(**a**,**b**) SEM, (**c**,**d**) HRTEM, and (**e**–**h**) HRTEM mapping images of BC@Co/Fe-5.

From the SEM images of BC@Co/Fe-5 shown in Figure 1a,b, the surface of BC@Co/Fe-5 is irregular and porous, and the metals are distributed nonuniformly. As shown in the HRTEM images of BC@Co/Fe-5 (Figure 1c,d), the metal particles have irregular shapes with sizes of 50–100 nm. The lattice fringe spacing of 0.203 nm (Figure 1d) corresponds to the (110) crystal plane of Fe [25]. Furthermore, an oxide film was observed on the outer layer of Fe [26]. The HRTEM mapping images of BC@Co/Fe-5 (Figure 1e–h) reveal that C and O have dense distributions, whereas Fe and Co have relatively disperse and uneven distributions.

**Figure 2:**
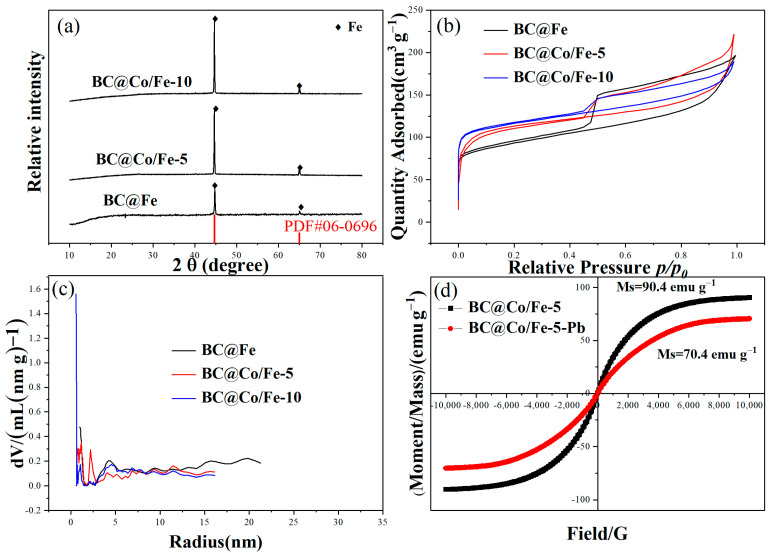
(**a**) XRD patterns, (**b**) N_2_ adsorption–desorption isotherms, and (**c**) pore size distributions of BC@Fe, BC@Co/Fe-5, and BC@Co/Fe-10. (**d**) Magnetisation curves of BC@Co/Fe-5 and BC@Co/Fe-5-Pb.

XRD was used to investigate the crystal structures of BC@Fe, BC@Co/Fe-5, and BC@Co/Fe-10 (Figure 2a). All these materials exhibit two relatively strong diffraction peaks at 2*θ* = 44.7° and 65.0°, which correspond to the (110) and (200) crystal planes, respectively, of Fe^0^ (PDF#06-0696) [27]. No characteristic Co peaks were observed, which was consistent with the relatively low Co contents of BC@Co/Fe-5 and BC@Co/Fe-10. Hysteresis loops were observed in the N_2_ adsorption–desorption isotherms of BC@Fe, BC@Co/Fe-5, and BC@Co/Fe-10 (Figure 2b). According to the IUPAC classification, the N_2_ adsorption–desorption isotherms are type IV and the hysteresis loops are type H2 [28]. The specific surface areas of BC@Fe, BC@Co/Fe-5, and BC@Co/Fe-10 are 464, 429, and 547 m^2^ g^−1^, respectively. The variation in the specific surface area is related to the type of aperture formed. The relatively high specific surface area of BC@Co/Fe-10 is related to its mainly microporous structure. The total pore volume and average pore size parameters of BC@Fe, BC@Co/Fe-5, and BC@Co/Fe-10 are listed in Table 1. The pore size distributions (Figure 2c) reveal that BC@Fe, BC@Co/Fe-5, and BC@Co/Fe-10 exhibit both microporous and mesoporous structures. Moreover, the proportion of micropores in the pore size distribution of BC@Co/Fe-10 is relatively high. The diameter of Pb^2+^ is 0.266 nm [29]. The average pore size parameters of BC@Co/Fe-10 is the smallest, about 2.65 nm, which inevitably increases the difficulty of Pb^2+^ diffusion. The magnetisation curves of BC@Co/Fe-5 and BC@Co/Fe-5-Pb (Figure 2d) revealed that the coercivity and remanence values of these materials are very small, which is typical for superparamagnetic materials [30]. The saturation magnetisations of BC@Co/Fe-5 and BC@Co/Fe-5-Pb are 90.2 and 70.4 emu g^−1^, respectively. As BC@Co/Fe-5-Pb has high magnetisation, after Pb^2+^ adsorption, the composite material can be easily collected and separated using a magnet.

### 2.2. Performance of BC@Fe and BC@Co/Fe-X

**Figure 3:**
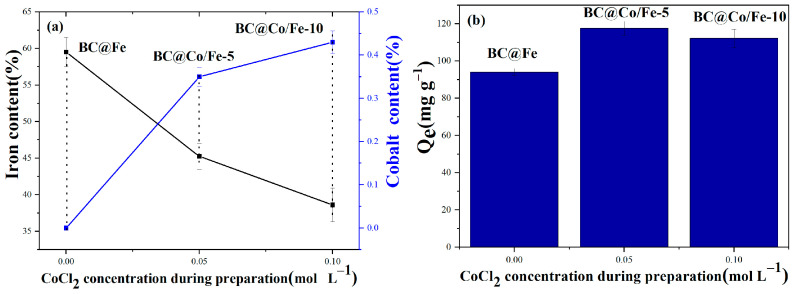
(**a**) Fe and Co contents and (**b**) Pb^2+^ adsorption properties of BC@Fe and BC@Co/Fe-X.

The Fe and Co contents of BC@Fe and BC@Co/Fe-X were determined following digestion, as shown in Figure 3a. BC@Fe has the highest Fe content of 59.5% and C and O contents of 32% and 5.2%, respectively. The content of Fe in BC@Fe is higher than that of zero-valent iron/biochar (Fe^0^-BC) prepared via carbothermal reduction using wood waste and iron mud as raw materials [31]. The Fe contents of BC@Co/Fe-5 and BC@Co/Fe-10 decrease significantly as the Co content increases, attributed to the complexing ability of different metal ions with alginate, which follows the sequence Fe^3+^ > Cu^2+^ > Co^2+^ [32]. Furthermore, Co^2+^ can significantly inhibit alginate cross-linking Fe^3+^. The Pb^2+^ adsorption properties of BC@Fe and BC@Co/Fe-X are compared in Figure 3b. The Pb^2+^ adsorption capacity of BC@Fe is the lowest. With increasing Co doping, the Pb^2+^ adsorption capacity of BC@Co/Fe-X first increases and then decreases. The decreased Pb^2+^ adsorption capacity of BC@Co/Fe-10 may be related to its lower Fe loading. The mass fractions of Fe and Co in BC@Co/Fe-5 are 45.3% and 0.35%, respectively.

### 2.3. Effect of pH on Pb^2+^ Adsorption

**Figure 4:**
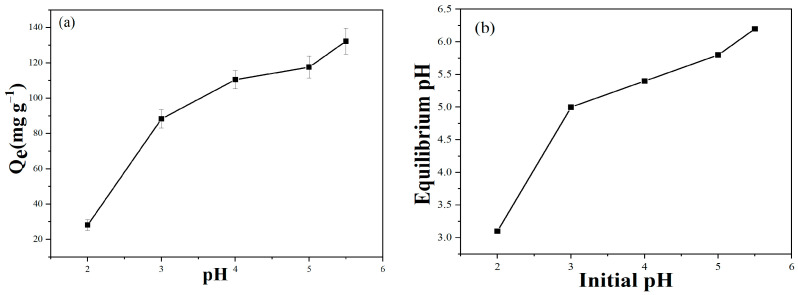
(**a**) Effect of initial pH on Pb^2+^ adsorption by BC@Co/Fe-5, and (**b**) the pH of the Pb^2+^ solution after adsorption by BC@Co/Fe-5.

The effect of pH on the Pb^2+^ adsorption performance of BC@Co/Fe-5 was investigated (Figure 4a). At pH 2, the Pb^2+^ adsorption capacity of BC@Co/Fe-5 is low, mainly because excess H^+^ can compete with Pb^2+^ for the adsorption sites. In addition, the higher H^+^ concentration at low pH favours the formation of Fe^2+^ from Fe^0^. The formation of iron oxides is difficult under low pH conditions, contributing to the low adsorption performance of BC@Co/Fe-5 [8]. As the H^+^ concentration decreases (pH 3–5.5), the Pb^2+^ adsorption capacity of BC@Co/Fe-5 increases [8]. After Pb^2+^ adsorption by BC@Co/Fe-5, the pH of the solution slightly increases (Figure 4b). Pb^2+^ forms hydrated ions at pH values higher than 6 and precipitates at pH values higher than 8. Therefore, Pb^2+^ adsorption experiments are generally conducted at pH values less than 7 [33]. Considering that Pb^2+^ remains in the form of ions and does not precipitate at pH values less than 5 [34], pH 5 was selected as optimal for Pb^2+^ adsorption in this study. 

### 2.4. Adsorption Kinetics 

**Figure 5:**
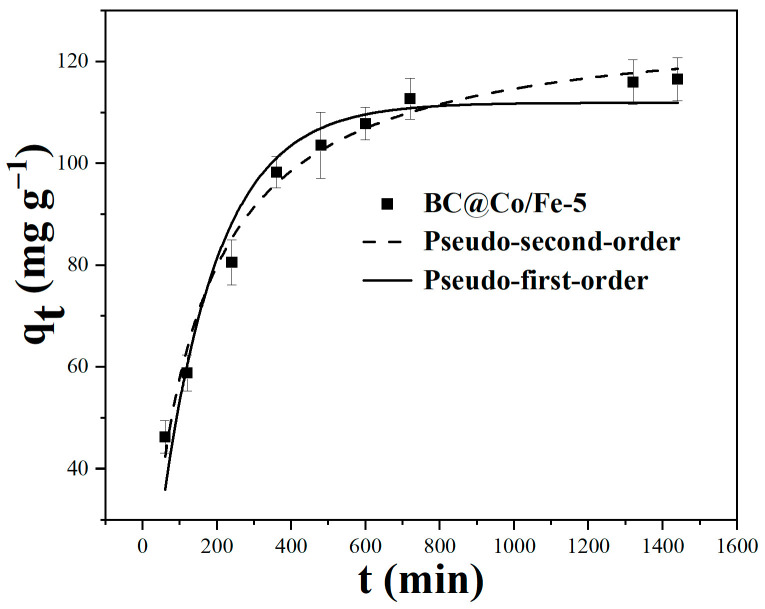
Pb^2+^ adsorption kinetics of BC@Co/Fe-5.

The kinetic data for Pb^2+^ adsorption by BC@CoFe-5 were fitted using pseudo-first-order and pseudo-second-order kinetic models. The obtained fitting parameters are summarised in Table 2, and Figure 5 shows the Pb^2+^ adsorption capacity of BC@Co/Fe-5 over time. As shown in Figure 5, the adsorption rate of Pb^2+^ on BC@Co/Fe-5 is very high over the first 6 h, likely because of a redox reaction between Fe and Pb^2+^ on the surface. Subsequently, the adsorption rate gradually decreases. During this stage, internal Fe may corrode to form Fe^2+^, which would convert Pb^2+^ into PbO.

As shown in Table 2, the correlation coefficient (*R*^2^ = 0.98) of the pseudo-second-order kinetic model is higher than that of the pseudo-first-order kinetic model (*R*^2^ = 0.95) for Pb^2+^ adsorption by BC@Co/Fe-5. However, the theoretical adsorption capacity (128.7 mg g^−1^) estimated from the pseudo-second-order equation was slightly higher compared to the experimental results (116.5 mg g^−1^). In addition, the theoretical adsorption capacity (111.9 mg g^−1^) obtained from the pseudo-first-order kinetic model was more consistent with the experimental results, suggesting that the rate-limiting step was governed via diffusion while the ions were mainly retained on the surface via chemical forces [35,36].

### 2.5. Adsorption Isotherms 

**Figure 6:**
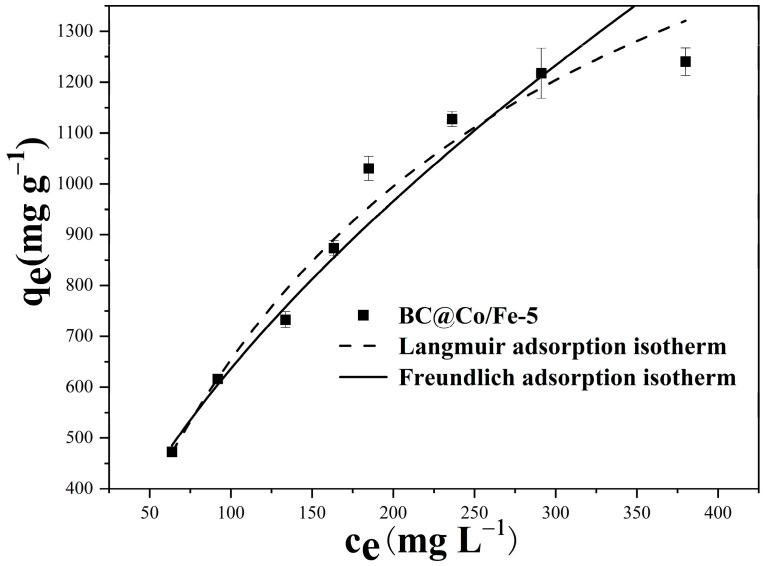
Fitted isotherms for Pb^2+^ adsorption by BC@Co/Fe-5.

The experimental data for Pb^2+^ adsorption by BC@Co/Fe-5 were fitted using the Langmuir and Freundlich models. The fitted curves for Pb^2+^ adsorption by BC@Co/Fe-5 are shown in Figure 6, with the fitting parameters listed in Table 3.

While the correlation coefficient (*R*^2^ = 0.98) of the Langmuir model is higher than that of the Freundlich model (*R*^2^ = 0.967) for Pb^2+^ adsorption by BC@Co/Fe-5, the theoretical adsorption capacity of the Langmuir model (2076.6 mg g^−1^) is higher than the actual adsorption capacity (1240 mg g^−1^), possibly because the Langmuir isotherm model assumes that active sites with identical energy exist on the surface of the adsorbent, which facilitates homogenous or surface-specific monolayer adsorption typical of chemical adsorption processes [37]. However, redox reactions and corrosion adsorption are the main adsorption mechanisms of Pb^2+^ by BC@Co/Fe-5. The Pb^2+^ adsorption process may be multilayer adsorption, confirmable via cyclic adsorption experiments. The values of *n*, which represent the favourability of the adsorption, were more than 1.0, indicating that the adsorption of Pb^2+^ by BC@Co/Fe-5 was favourable [38]. Table 4 compares the Pb^2+^ adsorption performance of BC@Co/Fe-5 and those of previously reported materials. Notably, BC@Co/Fe-5 exhibits superior Pb^2+^ adsorption performance.

### 2.6. Pb^2+^ Adsorption Mechanisms 

**Figure 7:**
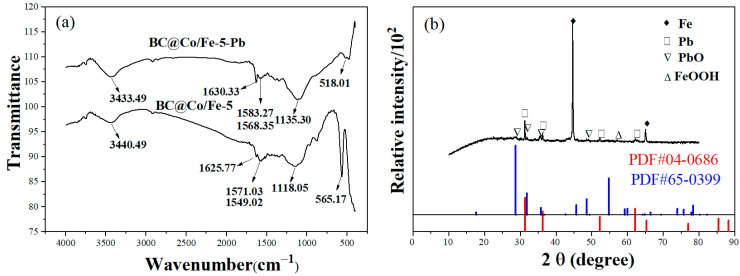
(**a**) FTIR spectra of BC@Co/Fe-5 before and after Pb^2+^ adsorption. (**b**) XRD pattern of BC@Co/Fe-5 after Pb^2+^ adsorption.

The FTIR spectra of BC@Co/Fe-5 before and after Pb^2+^ adsorption are shown in Figure 7a. For BC@Co/Fe-5 before Pb^2+^ adsorption, the strong, wide absorption peak at 3440.49 cm^−1^ corresponds to the stretching vibration of O–H [44], the absorption peak at 1625.77 cm^−1^ corresponds to the asymmetric stretching vibration of COO [44], the absorption peaks at 1571.03 and 1549.02 cm^−1^ are attributed to the vibration of the benzene ring skeleton [45], the absorption peak at 1118.05 cm^−1^ is attributed to the C–O–C stretching vibration [44], and the absorption peak at 565.17 cm^−1^ corresponds to the stretching vibration of Fe–O [44]. The Fe–O absorption peak appears because BC@Co/Fe-5 is oxidised to form ferrite during grinding. After Pb^2+^ adsorption by BC@Co/Fe-5, the characteristic absorption peaks at 3440.49, 1625.77, 1571.03, 1549.02, and 1118.05 cm^−1^ are blue-shifted to 3343.49, 1630.33, 1583.27, 1568.35, and 1135.30 cm^−1^, respectively [46]. This blue shift of the peaks corresponding to O–H, COO, the benzene ring skeleton, and C–O–C indicates the formation of a complex with Pb^2+^. The increased ionic volume weakened the stretching and bending vibrations of the functional groups, resulting in a blue shift [47]. 

The XRD pattern of BC@Co/Fe-5-Pb (Figure 7b) exhibits two strong diffraction peaks at 2*θ* = 44.7° and 65.0°, characteristic of Fe^0^ (PDF#06-0696) [27], indicating that the content of Fe^0^ in BC@Co/Fe-5 remains high after Pb^2+^ adsorption. In addition, new diffraction peaks appear at 2*θ* = 31.3°, 36.26°, 52.2°, and 62.1°, corresponding to the (111), (200), (220), and (311) crystal planes, respectively, of Pb (JCPDS no. 04-0686) [48]. The diffraction peaks at 2*θ* = 28.6°, 31.8°, 35.7°, and 48.5° correspond to the (101), (110), (002), and (112) crystal planes, respectively, of PbO (JCPDS no. 65-0399) [49]. The diffraction peak at 2*θ* = 57.1° corresponds to the (600) crystal plane of FeOOH (JCPDS no. 22-0353) [50]. These results indicate that Pb, PbO, and FeOOH formed on the surface of BC@Co/Fe-5 after Pb^2+^ adsorption.

**Figure 8:**
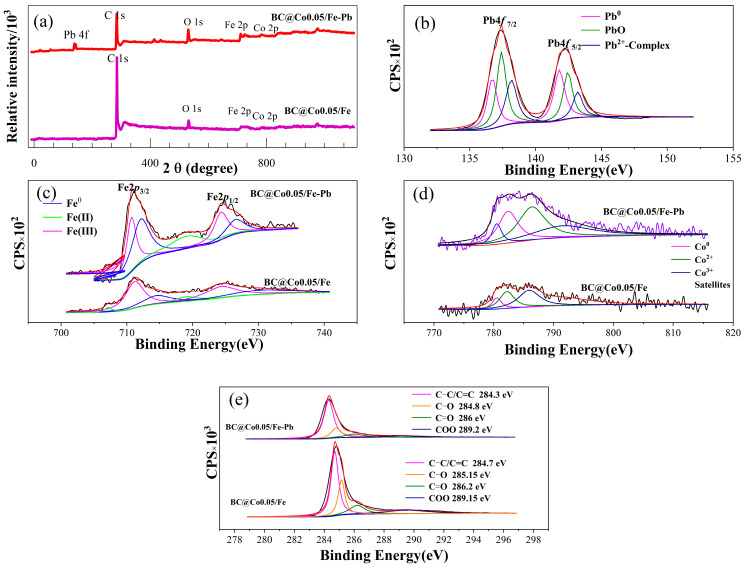
(**a**) XPS survey spectra of BC@Co/Fe-5 before and after Pb^2+^ adsorption. (**b**) Pb 4*f* XPS results for BC@Co/Fe-5 after Pb^2+^ adsorption. (**c**) Fe 2*p*, (**d**) Co 2*p*, and (**e**) C 1*s* XPS results for BC@Co/Fe-5 before and after Pb^2+^ adsorption.

The XPS survey spectra of BC@Co/Fe-5 before and after Pb^2+^ adsorption are shown in Figure 8a. An obvious Pb absorption peak appears in the XPS spectrum of BC@Co/Fe-5-Pb. The Pb XPS spectrum after Pb^2+^ adsorption by BC@Co/Fe-5 is shown in Figure 8b. The Pb 4*f*_7/2_ and Pb 4*f*_5/2_ peaks can each be fitted to three peaks [51]. The peak areas of the fitted peaks at 136.7 and 141.8 eV account for 35.8% of the total peak area, suggesting that some Pb^2+^ is reduced to Pb and adsorbed in this form [52]. The peak areas of the fitted peaks at 137.4 and 142.45 eV, which may correspond to PbO [52], account for 38.5% of the total peak area. The peak areas of the fitted peaks at 138.15 and 143.2 eV account for 25.7% of the total peak area, suggesting that Pb^2+^ could form a complex with active groups and be adsorbed as a Pb^2+^ complex [52].

The Fe XPS spectrum of BC@Co/Fe-5 (Figure 8c) exhibits Fe 2*p*_3/2_ and Fe 2*p*_1/2_ peaks. The fitted peaks at 707.19, 711.20, 714.50, 719.00, 724.30, and 730.40 eV correspond to Fe^0^ 2*p*_3/2_, Fe^2+^ 2*p*_3/2_, Fe^3+^ 2*p*_3/2_, Fe^0^ 2*p*_1/2_, Fe^2+^ 2*p*_1/2_, and Fe^3+^ 2*p*_1/2_, respectively [53]. Fitting of the Fe 2*p*_3/2_ and Fe 2*p*_1/2_ peaks of BC@Co/Fe-5-Pb led to peaks at 710.7, 712.1, 719.00, 724.30, and 726.5 eV, corresponding to Fe^2+^ 2*p*_3/2_, Fe^3+^ 2*p*_3/2_, Fe^0^ 2*p*_1/2_, Fe^2+^ 2*p*_1/2_, and Fe^3+^ 2*p*_1/2_, respectively. The disappearance of the Fe^0^ 2*p*_3/2_ peak at 707.19 eV indicates that Fe is consumed during Pb^2+^ adsorption.

The Co XPS spectrum of BC@Co/Fe-5 is shown in Figure 8d. Fitting yielded four peaks at 780.5, 782.2, 785.9, and 791.5 eV, corresponding to Co^0^, Co^2+^, Co^3+^, and satellites, respectively. Fitting of the Co XPS spectrum of BC@Co/Fe-5-Pb also afforded four peaks [54]; the peaks at 780.5, 782.4, 786.4, and 791.5 eV correspond to Co^0^, Co^2+^, Co^3+^, and satellites, respectively [54,55]. The changes in the binding energies of the characteristic Fe and Co peaks before and after adsorption may be due to the reactions with Pb^2+^. The Fe morphologies in BC@Co/Fe-5 revealed by the XPS (Figure 8c) and XRD (Figure 2a) analyses differ slightly. As the depth of the XPS analysis is limited to within 5 nm of the surface of the material, the results are affected by Fe oxidation that occurs on the surface layer during transportation and testing. In contrast, the depth of the XRD analysis is generally several to dozens of micrometres, and surface oxidation has little effect on the results.

The C XPS spectrum of BC@Co/Fe-5 (Figure 8e) exhibits major absorption peaks corresponding to C–C/C=C (284.70 eV), C–O (285.15 eV), C=O (286.2 eV), and COO (289.15 eV) [56]. The change in the binding energies of C–C/C=C, C–O, C=O, and COO after Pb^2+^ adsorption by BC@Co/Fe-5 suggests that these functionalities form complexes with Pb^2+^ during the adsorption process, consistent with the FTIR spectroscopy results (Figure 7a). 

**Figure 9:**
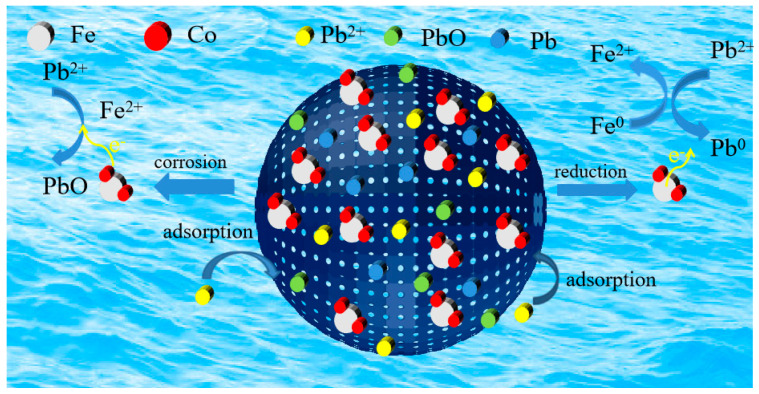
Mechanism of Pb^2+^ adsorption by BC@Co/Fe-5.

The FTIR, XRD, and XPS results before and after Pb^2+^ adsorption by BC@Co/Fe-5 indicate that most Pb^2+^ was converted into Pb and PbO, as shown in Equations (1)–(5), while the remainder was adsorbed as Pb^2+^ [57]. The proposed adsorption mechanism is illustrated in Figure 9.

(1) $$2Fe^{0}+3Pb^{2+}+4H_{2}O\overset{Co}{\to }3Pb^{0}+2FeOOH+6H^{+}$$

(2) $$2Fe^{0}+O_{2}+4H^{+}\to 2Fe^{2+}+2H_{2}O$$

(3) $$Fe^{2+}+H_{2}O\to FeOH^{+}+H^{+}$$

(4) $$FeOH^{+}+Pb^{2+}\to PbOH^{+}+Fe^{2+}$$

(5) $$PbOH^{+}\to PbO+H^{+}$$

### 2.7. Recyclability of BC@Co/Fe-5

**Figure 10:**
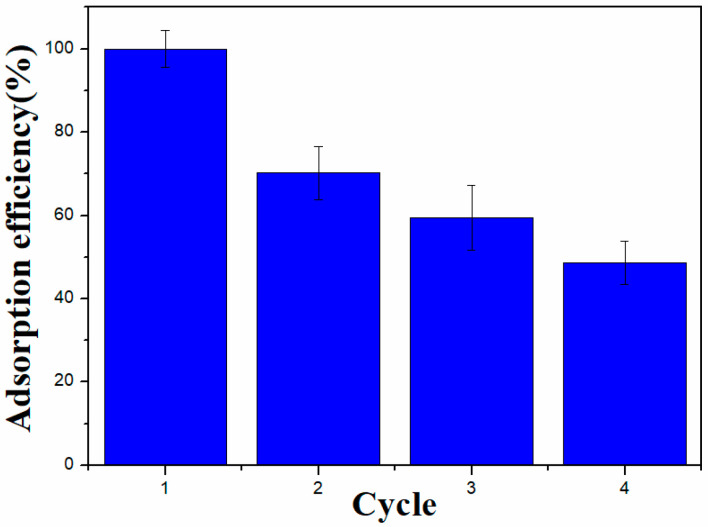
Recyclability of BC@Co/Fe-5.

Figure 10 shows the Pb^2+^ adsorption performance of BC@Co/Fe-5 over four cycles. The adsorption capacities of BC@Co/Fe-5 for Pb^2+^ in the second, third, and fourth cycles are 29.8%, 39.6%, and 51.3%, respectively, lower than the initial adsorption capacity. Thus, BC@Co/Fe-5 maintains 48.7% of the initial adsorption capacity after four cycles. The cyclic performance likely deteriorates because the adsorption of Pb^2+^ by Co and Fe inside BC@Co/Fe-5 is relatively difficult. The newly added high concentration of Pb^2+^ increases the chance of contact between Fe and Pb^2+^ and the adsorption amount of Pb^2+^. In addition, the presence of H^+^ in the Pb^2+^ solution during subsequent cycles can promote the corrosion of Fe inside BC@Co/Fe-5 to form Fe^2+^, which can participate in Pb^2+^ adsorption [58].

### 2.8. Pb^2+^ Removal from Contaminated Wastewater and Soil

**Figure 11:**
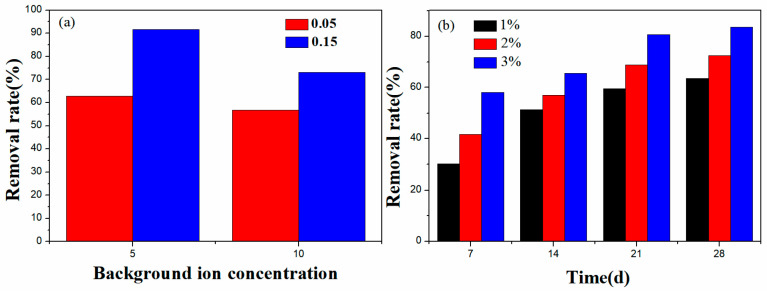
Remediation of (**a**) simulated Pb^2+^-containing wastewater and (**b**) simulated Pb^2+^-contaminated soil using BC@Co/Fe-5.

The Pb^2+^ adsorption results for BC@Co/Fe-5 in simulated wastewater are shown in Figure 11a. Upon the addition of 0.05 and 0.15 g BC@Co/Fe-5, the Pb^2+^ removal rates reach 62.9% and 91.7%, respectively, under the condition of background ions at five times their standard concentrations. When the concentrations of the background ions are increased to ten times their standard values, the Pb^2+^ removal rates of 0.05 and 0.15 g BC@Co/Fe-5 decrease to 56.7% and 73.2%, respectively. 

The results for the remediation of simulated Pb^2+^-contaminated soil are shown in Figure 11b. When 1, 2, or 3 wt.% BC@Co/Fe-5 is added to the soil, the Pb^2+^ content decreases by 30.2%, 41.7%, and 58%, respectively, after 7 d, and by 59.6%, 68.9%, and 80.7% after 21 d. The remediation effect does not change significantly after 28 d relative to that after 21 d, suggesting that the system was close to equilibrium after 21 d. Upon increasing the BC@Co/Fe-5 dosage from 1 wt.% to 3 wt.%, the remediation effect on Pb^2+^-contaminated soil increases gradually.

## 3. Materials and Methods

### 3.1. Materials 

The materials used in this study included SA, FeCl_3_·6H_2_O, and CoCl_2_ (AR, Aladdin, Shanghai, China); NaOH (AR, Shanghai Yi En Chemical Technology Co., Ltd., Shanghai, China); PbCl_2_ and NaNO_3_ (AR, Shanghai Baishun Biotechnology Co., Ltd., Shanghai, China); Pb and Co standard solutions (China National Academy of Metrology Sciences, Beijing, China); and pH buffer reagent (Apure, Shanghai, China).

### 3.2. Preparation of BC@Co/Fe-X

In a beaker, 30 g SA was dissolved in 1 L of distilled water by stirring (B90-SH, Guangzhou Gangran Electromechanical Equipment Co., Ltd., Guangzhou, China). The SA solution was added dropwise to a solution containing 0.3 mol L^−1^ FeCl_3_ and 0.05 or 0.1 mol L^−1^ CoCl_2_, with the mixture allowed to stand for 24 h to undergo cross-linking. The obtained gel was recovered via filtration, with metal ions on the surface removed by washing with distilled water (6–8 times). The gel was dried in a vacuum oven (JHG-9053A, Shanghai Jinghong Experimental Equipment Co., Ltd., Shanghai, China) at 60 °C and then at 100 °C for 3 h. The dried SA-Fe^3+^/Co^2+^ gel was placed in a crucible and inserted into the quartz tube of a tube furnace (OTF1200X, Hefei Kejing Material Technology Co., Ltd., Hefei, China). After evacuation for 15 min, high-purity nitrogen was introduced at a flow rate of 200 mL min^−1^ for 20 min. Under nitrogen at a flow rate of 100 mL min^−1^, the temperature was increased to 200 °C at a heating rate of 5 °C min^−1^ and then to 900 °C at a heating rate of 10 °C min^−1^. After pyrolysis at 900 °C for 3 h, the furnace was cooled to room temperature at a cooling rate of 10 °C min^−1^. The prepared Co/Fe biochar composites (BC@Co/Fe-X, where X = 5 or 10 represents the CoCl_2_ concentration of 0.05 or 0.1 mol L^−1^) were stored under vacuum [36]. 

### 3.3. Characterisation

Scanning electron microscopy (SEM) images were obtained using a JSM-7400F microscope to observe the basic morphology of the as-prepared samples. Transmission electron microscopy (TEM), high-resolution TEM (HRTEM), and elemental mapping analyses were conducted on a JEM-2100Plus microscope (JEOL Ltd., Akishima-shi, Japan). X-ray diffraction (XRD) measurements were performed using an UItima IV diffractometer (Rigaku, Tokyo Metropolis, Japan) with a Cu Kα light source (*λ* = 0.15418 nm) at an operating voltage of 40 kV and an operating current of 10 mA in the scanning range of 2*θ* = 10–80°. Magnetisation curves were obtained using a SQUID-VSM magnetic measuring system (Quantum Design, San Diego, CA, USA). N_2_ adsorption–desorption isotherms were obtained using a Tristar II 3020 surface area and porosity analyser (Micromeritics, Atlanta, GA, USA). X-ray photoelectron spectroscopy (XPS) measurements were conducted using an ESCALAB 250Xi spectrometer (Thermo Fisher, Waltham, MA, USA) with an Al Kα light source (h*v* = 1486.6 eV) at a power of 150 W and a 500 µm spot energy analyser at a transmission energy of 30 eV. Fourier transform infrared (FTIR) spectra were recorded using a Thermo Fisher 6700 FTIR spectrometer (Thermo Fisher, Waltham, MA, USA). The elemental analysis of carbon and oxygen was conducted using a Vario EL cube elemental analyser (Elementar, Frankfurt, Germany).

### 3.4. Batch Sorption Experiments

To compare the Pb^2+^ adsorption performance of different materials, 0.05 g of BC@Fe, BC@Co/Fe-5, or BC@Co/Fe-10 was added to 100 mL of a 100 mg L^−1^ Pb^2+^ solution (pH 5) in a conical flask. The conical flasks were placed in a constant temperature shaker at 25 °C for 24 h. To compare the effect of pH on Pb^2+^ adsorption by BC@Co/Fe-5, 0.05 g BC@Co/Fe-5 was added to 100 mL of a Pb^2+^ solution (100 mg L^−1^) with pH values of 2, 3, 4, 5, or 5.5 in a conical flask. The conical flasks were placed in a constant temperature shaker at 25 °C for 24 h. To analyse the kinetics of Pb^2+^ adsorption by BC@Co/Fe-5, 0.05 g BC@Co/Fe-5 was added to 100 mL of Pb^2+^ solution (100 mg L^−1^) in a conical flask [9]. The conical flask was placed in a constant temperature shaker at 25 °C. At fixed times (1–24 h), 1 mL of the Pb^2+^ solution was removed and diluted for analysis [59]. For thermodynamic experiments, 0.05 g BC@Co/Fe-5 was added to 100 mL of a Pb^2+^ solution (pH 5) with concentrations of 300–1000 mg L^−1^ in a conical flask. The conical flasks were placed in a constant temperature shaker at 25 °C for 24 h, and then 1 mL of the Pb^2+^ solution was removed and diluted for analysis [59]. To investigate the recyclability, 0.05 g BC@Co/Fe-5 was added to 100 mL of a Pb^2+^ solution (100 mg L^−1^), with the mixture shaken at 25 °C for 24 h. After the adsorption process was complete, the solution was removed. Then, fresh Pb^2+^ solution was added, with the mixture shaken again for 24 h under the same conditions [58]. The adsorption performance of BC@Co/Fe-5 was investigated over four cycles.

The data for the kinetic adsorption of Pb^2+^ by BC@Co/Fe-5 were fitted using pseudo-first-order and pseudo-second-order kinetic models (Equations (6) and (7), respectively) [60]. From a mathematical point of view, the pseudo-first-order model well describes the kinetic processes limited from beginning to end by film diffusion at the phase boundary. It also describes the final stages (the so-called regular modes) of particle diffusion processes with a good approximation [2]. The pseudo-second-order model explains the chemisorption of heavy metal ions onto adsorbents [61].

(6) $$q_{t}=q_{e}(1-e^{\;k_{1}t})$$

(7) $$q_{t}=\frac{k_{2}{q_{e}}^{2}t}{1+k_{2}q_{e}t}$$

where q_t_ is the adsorption capacity at time t (mg g^−1^), q_e_ is the adsorption capacity at equilibrium (mg g^−1^), k_1_ is the first-order rate constant (min^−1^), k_2_ is the second-order rate constant (g mg^−1^ min^−1^), and t is time (min). 

The thermodynamic data for Pb^2+^ adsorption by BC@Co/Fe-5 were fitted using the Langmuir and Freundlich models (Equations (8) and (9), respectively) [62].

(8) $$q_{e}=\frac{q_{m}K_{L}c_{e}}{1+K_{L}c_{e}}$$

(9) $$q_{e}=K_{F}{c_{e}}^{1/n}$$

where q_e_ is the adsorption capacity at adsorption equilibrium (mg g^−1^), q_m_ is the adsorption capacity at saturation (mg g^−1^), c_e_ is the concentration of Pb^2+^ in solution at adsorption equilibrium (mg L^−1^), K_L_ is the Langmuir adsorption constant (L mg^−1^), and K_F_ is the Freundlich adsorption constant (mg^1−n^ L^n^ g^−1^). 

### 3.5. Pb^2+^ Adsorption by BC@Co/Fe-5 in Simulated Samples

To investigate the ability of BC@Co/Fe-5 to remove Pb^2+^ from sewage, Class V water from the China groundwater standard (GB/T 14848-2017) [63], containing 350 mg L^−1^ Cl^−^, 350 mg L^−1^ SO_4_^2−^, 350 mg L^−1^ CO_3_^2−^, 300 mg L^−1^ NO_3_^−^, 1.5 mg L^−1^ Cu^2+^, and 5 mg L^−1^ Zn^2+^, was used as the background [64]. The Pb^2+^ (100 mg L^−1^) removal efficiency was investigated by adding 0.05 or 0.15 g BC@Co/Fe-5 to 100 mL of a background solution containing Cl^−^, SO_4_^2−^, CO_3_^2−^, NO_3_^−^, Cu^2+^, and Zn^2+^ at concentrations five and ten times higher than those in the Class V groundwater standard.

To investigate the ability of BC@Co/Fe-5 to remove Pb^2+^ from the soil, surface soil (0–20 cm) was collected from Guilinyang Farm, Haikou City, and dried in the shade. The soil was crushed using a soil crusher and passed through a 60-mesh sieve. Simulated contaminated soil with a Pb content of 800 mg kg^−1^ was prepared by adding a PbCl_2_ solution to the soil. The contaminated soil was dried in the shade, crushed, and passed through a 60-mesh sieve. Then, 25 g simulated contaminated soil was mixed with BC@Co/Fe-5 (1, 2, and 3 wt.%), and deionised water was added to achieve a soil moisture content of 75%. The soil was stored in the dark and weighed every 2 d, after which water was added to maintain a constant soil moisture content. Soil samples were removed and freeze-dried on days 7, 14, 21, and 28 [65]. 

The Pb^2+^ content was measured using toxicity characteristic leaching procedure extraction and atomic absorption methods, as regulated by the United States Environmental Protection Agency (1992); these methods have been widely used to analyse the toxicity characteristics of toxic elements in polluted soil [66].

## 4. Conclusions

In this study, well-dispersed and polyporous BC@Co/Fe-X was prepared via carbothermal reduction. Co-doping improved the Pb^2+^ adsorption capacity of BC@Co/Fe-X, with the highest adsorption capacity observed for BC@Co/Fe-5, which had Fe and Co mass fractions of 45.3% and 0.35%, respectively. Notably, such a low Co content is not harmful to the environment. The mechanism of Pb^2+^ adsorption on BC@Co/Fe-5 comprised the following processes: (i) Co/Fe reduction of Pb^2+^ to Pb^0^, (ii) Co/Fe corrosion to form Fe^2+^ and fix Pb^2+^ as PbO, and (iii) Pb^2+^ adsorption by the Co/Fe biochar. The redox reaction and corrosion adsorption are the main adsorption mechanisms. Kinetic fitting to the result indicated that the rate-limiting step was governed via diffusion while the ions were mainly retained on the surface via chemical forces. BC@Co/Fe-5 maintains 48.7% of its initial Pb^2+^ adsorption capacity after four cycles. Furthermore, the acidic and high concentration of Pb^2+^ facilitated the iron corrosion continuously generated, resulting in highly effective adsorption of Pb^2+^. Isotherms fitting to the result indicated that the Pb^2+^ adsorption process may be multilayer adsorption, confirmable via cyclic adsorption experiments. Reasonable Pb^2+^ removal effects were observed in simulated wastewater, with 0.15 g BC@Co/Fe-5 achieving Pb^2+^ removal rates of up to 91.7% and 73.2% under the condition of background ions at five and ten times their standard concentrations, respectively. For soil remediation, the addition of 3 wt.% BC@Co/Fe-5 reduced the Pb^2+^ content of the soil by 83.5% after 28 d. Overall, BC@Co/Fe-5 is a cost-effective and environmentally sustainable composite, holding promise for the remediation and treatment of Pb^2+^ pollution.

## Figures and Tables

**Table 1:** Textural properties of BC@Fe, BC@Co/Fe-5, and BC@Co/Fe-10.

Sample	Surface Area (m^2^ g^−1^)	Total Pore Volume (cm^3^ g^−1^)	Average Pore Size Parameters (nm)
BC@Fe	464	0.303	3.53
BC@Co/Fe-5	429	0.294	3.21
BC@Co/Fe-10	547	0.343	2.65

**Table 2:** Constants and correlation coefficients for the Pb^2+^ adsorption kinetics of BC@Co/Fe-5.

Sample	Pseudo-First-Order Model						Pseudo-Second-Order Model					
BC@Co/Fe-5	*q*_e,cal_ (mg g^−1^)	SE_x_	*k*_1_ (10^−3^ min^−1^)	*R* ^2^	RSS	Reduced chi-squared	*q*_e,cal_ (mg g^−1^)	SE_x_	*k*_2_ (10^−5^ g mg^−1^ min^−1^)	*R* ^2^	RSS	Reduced chi-squared
	111.9	3.09	6.48	0.95	17.2	2.5	128.7	2.92	6.36	0.98	6.0	0.87

**Table 3:** Isotherm fitting parameters for Pb^2+^ adsorption by BC@Co/Fe-5.

Sample	Langmuir						Freundlich					
BC@Co/Fe-5	*q*_m,cal_ (mg g^−1^)	SE_x_	*K*_L_ (L mg^−1^)	*R* ^2^	RSS	Reduced chi-squared	*n*	*K*_F_ (mg^1−n^ L^n^ g^−1^)	SE_x_	*R* ^2^	RSS	Reduced chi-squared
	2076.6	155.8	0.0046	0.98	43.3	7.22	1.66	39.76	7.63	0.967	91.2	15.20

**Table 4:** Pb^2+^ adsorption properties of various materials.

Adsorbent	pH	Concentration Range (mg L^−1^)	*q*_m_ (mg g^−1^)	Ref.
Modified chitosan hydrogel	5	100–1100	420.98	[3]
Amino-carboxyl cellulose	5	100–1000	117.6	[6]
Analcime-activated carbon composite	5.5	100	125.57	[4]
Sulfhydryl-rich β-cyclodextrin polymers	-	-	604.64	[39]
Alkali and alkaline earth metal-rich biochar	5.5	10–4000	226.64	[40]
Mesoporous zeolite-A/reduced graphene oxide	6.5	-	416.7	[41]
Carboxymethyl cellulose-nZVI	6	100–1000	1376	[8]
Geopolymer-based zeolite microspheres	5	100–600	529.7	[34]
Carboxymethylcellulose/graphene oxide + Fe 18%	6	55–1050	1850	[2]
Kaolinite nanotubes	6	25–350	1428	[42]
Polyaspartic acid/carboxymethyl salix psammophila hydrogel	5.5	2000–10,000	1954	[43]
BC@Co/Fe-5	5	300–1000	1240	This study

## Data Availability

Data are contained within the article.

## Abbreviations

BC@Co/Fe-X	Co/Fe biochar composite
CBC	corn cob biochar
c_e_	concentration at adsorption equilibrium (mg L^−1^)
FTIR	Fourier transform infrared
HRTEM	high-resolution transmission electron microscopy
k_1_	first-order rate constant (min^−1^)
k_2_	second-order rate constant (g mg^−1^ min^−1^)
K_L_	Langmuir adsorption constant (L g^−1^)
K_F_	Freundlich adsorption constant (mg g^−1^)
nZVI	nano-zero-valent iron
q_e_	adsorption capacity at equilibrium (mg g^−1^)
q_m_	adsorption capacity at saturation (mg g^−1^)
q_t_	adsorption capacity at time *t* (mg g^−1^)
SE_x_	standard error
RSS	residual sum of squares
SA	sodium alginate
SEM	scanning electron microscopy
t	time (min)
XPS	X-ray photoelectron spectroscopy
XRD	X-ray diffraction
